# Bibliometric Review and Research Evolution on the Durability of LC3 Cement

**DOI:** 10.1155/tswj/4832631

**Published:** 2025-12-08

**Authors:** Ayman Shamseldein, Rabee Shamass, Xiangming Zhou

**Affiliations:** ^1^ Structural Engineering Department, Faculty of Engineering, Shams University, Cairo, Egypt; ^2^ Department of Civil and Environmental Engineering, College of Engineering, Design and Physical Sciences, Brunel University of London, London, UK

**Keywords:** bibliometric analysis, cluster analysis, durability, limestone calcined clay, VOSviewer

## Abstract

The use of sustainable materials in the construction industry has recently gained significant attention. Limestone calcined clay cement (LC3) is arising as a viable alternative to ordinary Portland cement (OPC). However, few studies were found focusing on the durability of LC3. This study conducts a bibliometric analysis to assess the evolution of LC3 durability research using data extracted from the Scopus and Web of Science databases covering the period 2017–2025. A total of 21 articles were analyzed using Microsoft Excel and VOSviewer to evaluate publication trends, author productivity, journal sources, and geographical distribution. Results indicate a growing research interest, with publications peaking in 2023–2024. India, China, and Switzerland lead the field. Despite this progress, research gaps persist regarding LC3 performance under freeze–thaw cycles, alkali–silica reaction, elevated temperatures, and combined environmental exposures. Addressing these gaps through international collaboration and comprehensive testing is vital for advancing LC3′s global adoption in sustainable construction.

## 1. Introduction

Portland cement concrete is the most widely used man‐made material and is unrivaled for its versatility and durability [1]. Accordingly, it is an indispensable ingredient for modern civilization used in the construction of civil engineering and architectural structures. Like many manufacturing processes, Portland cement production is not without detriment to the environment with respect to the energy‐intensive nature of its production and the inherent CO_2_ emissions. Calcination is the primary source of CO_2_ emissions in addition to the CO_2_ that is released through burning fossil fuels for heating the kiln. The global cement industry accounts for around 7% of global CO_2_ emissions [2]. The UN Intergovernmental Panel on Climate Change in COP27 [3] held in Sharm El Sheikh recommended that CO_2_ emissions must be reduced within 45% by 2030, to enable compliance with the criterion of not exceeding 2°C temperature by 2050. With respect to embodied energy and CO_2_ emissions, the state of the art for Portland cement production is approaching the point where existing production technologies are so refined in their energy efficiency that they can only minimally influence the desired emission outcomes. Blended cement is one of the major Portland cement alternatives to meet high concrete demand due to urbanization and growth. Adoption of blended cements can result in numerous environmental advantages, including the beneficial and effective incorporation of recycled and residual materials, and reductions in the embodied energy and CO_2_ emissions associated with Portland cement production [4]. The impetus to rapid growth in the production of blended cements in many countries came as a result of their energy saving and clinker saving potential. Also, in certain respects, blended cements perform better than ordinary Portland cement. Recently, the production of blended Portland cements, slag cements, and pozzolanic cements in Europe and Asia has exceeded the production of pure Portland cements. The blended cement resulted in reduction of at least 25% in CO₂ emissions, fuel consumption, and cost [5]. This success has encouraged cement factories to conduct further research to develop even more sustainable and cost‐effective cement solutions. Therefore, it is recommended to continue research efforts to explore the implementation of limestone calcined clay cement (LC3) [6]. This new cement can reduce CO₂ emissions by 30%–50% [6], as 50% of the clinker is replaced with a mixture of calcined clay and limestone. Calcined clay is natural clay heated to nearly 800°C [7], which is significantly lower than the typical cement kiln temperature, making the process more energy efficient and environmentally friendly. The mechanical properties of LC3 have demonstrated superior strength compared with OPC and blended cements. Several researchers have successfully produced LC3 with higher compressive strength using the same mixing ratios as those for OPC [8, 9]. Although several studies have been conducted on the mechanical properties of LC3, few have focused on its durability. Durability is a crucial aspect as it helps reduce CO_2_ emissions by extending the lifespan of structures and preventing the need for costly reconstruction or extensive repair and strengthening. Therefore, this article aims to highlight the durability of LC3 and perform a bibliometric analysis to examine the authors, countries, and number of publications on the topic. It is important to note that the properties of LC3 can vary depending on the region, as the source of clay differs, making it essential to conduct studies for each clay source worldwide with different compositions. This study will identify which regions have conducted sufficient research and which still require further investigation. According to Manosa et al. [10], bibliometric analyses have been applied in various research fields, including econometrics, management, statistics, ecology, waste management, production management, renewable energy, and thermal energy storage technologies [11]. The use of bibliometric analyses has also extended to the construction and building research domain. In 2014, Cañas‐Guerrero et al. published a comprehensive bibliometric analysis of construction and building technology [12]. Since then, several studies have focused on specific construction materials: Det Udomsap and Hallinger investigated sustainable construction [13], Chang et al. studied publications related to asphalt and pavement [14], Afgan and Bing conducted a review on the use of phase changing materials (PCMs) in building applications [15], and Yang et al. presented recent publications on geopolymer composites [16].

The primary aim of this research is to carry out bibliometric analysis on the durability of the new low‐carbon cement, LC3, by offering an overview of the research and development in this area. Additionally, this study seeks to find the gap in the durability studies of LC3. This can encourage researchers to carry out more researches in the durability of LC3.

## 2. Methodology

**Figure 1:**
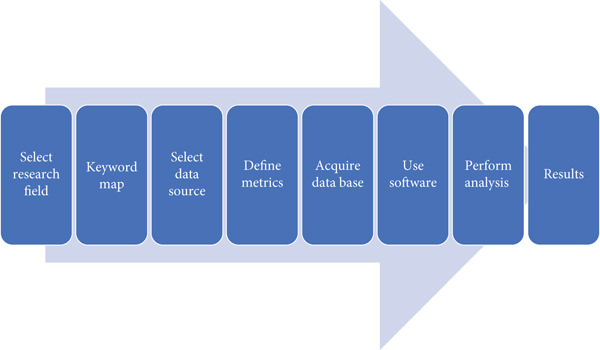
Methodology adopted for the bibliometric analysis of LC3 durability research.

The approach used in this bibliometric study is based on the methodology used by Manosa et al. [10], which has been extensively applied in numerous bibliometric analyses [11, 17]. The methodology is shown in Figure 1. To define the appropriate terms for searching publications in this research field, several keywords and phrases were selected. The primary keywords (search formula) included: “limestone,” “calcined clay,” and “durability.” To choose a valid publication, all the keywords in the same row must appear in the document′s title, author keywords, or abstract.

After defining the keywords, the next step was to choose the most appropriate data sources. Web of Science (WoS) from Clarivate Analytics and Scopus from Elsevier were selected due to their reliable and accessible publications [18]. Additionally, only scientific papers were included in the results by applying filters to both data sources. Once the bibliometric database was acquired, it was processed using MS Excel. Furthermore, VOSviewer software was used to analyze the raw data extracted from Scopus and WoS.

### 2.1. Inclusion and Exclusion Criteria

• Inclusion: Publications focusing on LC3 or blended cements where durability properties (e.g., chloride ingress, carbonation, alkali‐silica reaction [ASR], sulfate attack, freeze–thaw, or permeability) were investigated.
• Exclusion: Papers dealing solely with mechanical properties, hydration kinetics, or life cycle assessments without experimental or analytical discussion of durability.

### 2.2. Time Frame Justification

The time window 2017–2025 was selected because the first LC3 durability study indexed in Scopus appeared in 2017. Earlier years yielded no relevant results, confirming that research in this area began recently and remains rapidly evolving.

### 2.3. Data Analysis and Visualization

The bibliometric data (21 articles) were processed in Microsoft Excel for descriptive statistics and VOSviewer (version 1.6.20) for mapping co‐authorship, country collaboration, and keyword co‐occurrence networks. In VOSviewer, a minimum keyword occurrence threshold of two was applied to focus on relevant and frequently used terms. The association strength normalization method and Lin‐Log/modularity clustering technique were used to generate clear, nonoverlapping visualizations of research clusters.

## 3. Results

### 3.1. Publication and Source Evolution

**Figure 2:**
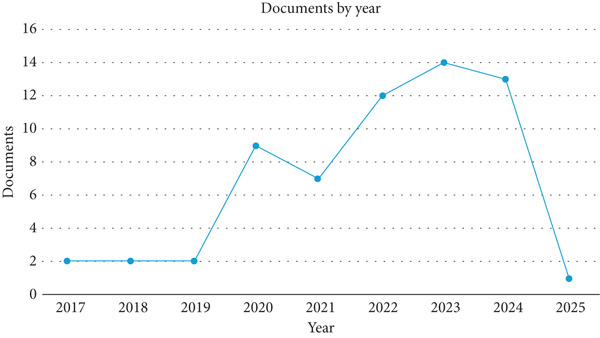
Annual number of publications on the durability of LC3 indexed in the Scopus database (2017–2025).

**Figure 3:**
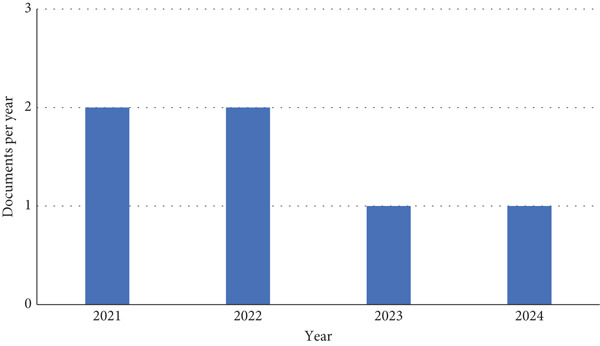
Annual number of publications on the durability of LC3 indexed in the Web of Science (WoS) database (2021–2024).

Figures 2 and 3 show the published articles on Scopus and WoS in the last 10 years, respectively. The two figures illustrate the number of research articles on the durability of LC3 published over different time periods: Scopus from 2017 to 2025 and WoS from 2021 to 2024. The period in the graphs was selected based on the available publications. Therefore, no publication was found before this period. By comparing the trends in these databases, we can gain insight into the differences in publication patterns, coverage, and research focus on LC3 durability during the overlapping years and beyond.

The Scopus graph, a line graph spanning 2017–2025, shows a low and stable number of publications from 2017 to 2019, with around two documents per year. A significant increase occurs in 2020, with the number of documents rising to eight, followed by a peak of 12 in 2021, a slight dip to 10 in 2022, and a rise to 14 in 2023. The number remains high at 14 in 2024 before dropping sharply to two in 2025, likely due to incomplete data as the current date is March 2025, capturing only the first quarter of the year. This trend indicates a gradual buildup of interest in LC3 durability research, with a notable surge from 2020 onward, peaking in 2023–2024.

In contrast, the WoS histogram, covering 2021–2024, shows a more concentrated trend. In 2021 and 2022, the number of documents is at its highest, with around two documents each year, followed by a decline to one document per year in 2023 and 2024. This pattern suggests a peak in high‐impact LC3 durability research in 2021–2022, with a subsequent drop in publication activity. WoS, known for its selective indexing of high‐impact journals, likely publishes fewer but more significant studies, focusing on quality over quantity.

Focusing on the overlapping years (2021–2024), Scopus shows a higher number of publications compared to WoS. In 2021, Scopus reports 12 documents, whereas WoS has only two. In 2022, Scopus has 10 documents, again compared with WoS ′s two. By 2023, Scopus peaks at 14 documents, whereas WoS drops to one, and in 2024, Scopus maintains 14 documents, whereas WoS remains at one.

The trends also reveal differences in the trajectory of research interest. Scopus shows a sustained increase in LC3 durability research, peaking in 2023–2024, which aligns with the material′s growing adoption in industry. WoS, however, shows a peak earlier in 2021–2022, suggesting that the most impactful studies may have been published during this period, with a decline in high‐impact output afterward. In addition, the bibliometric analysis shows that the research in the durability of LC3 is still a new and emerging research interest.

Table 1 shows the most cited publication from Scopus and WoS. The bibliometric analysis of highly cited research on LC3 highlights key contributions to its mechanical properties and durability performance. Table 1 reveals that Dhandapani et al. [8] holds the highest citation count (397 citations), reflecting the growing interest in LC3′s performance in terms of oxygen permeability, chloride penetration, resistivity, and water sorptivity. This study, published in *Cement and Concrete Research*, serves as a foundational work in durability assessment. The results demonstrate that the LC3 binder performs better than other binders in producing durable concrete, particularly in chloride‐rich environments. This superior performance is mainly due to the more compact and denser microstructure of the system with the LC3 binder compared to OPC.

**Table 1:** Most cited articles.

**Title**	**Authors**	**Year**	**Journal**	**Citations**	**Durability tests conducted**	**Reference**
Mechanical Properties and Durability Performance of Concretes With Limestone Calcined Clay Cement (LC3)	Dhandapani, Y.; Sakthivel, T.; Santhanam, M.; Gettu, R.; and Pillai, R. G.	2018	*Cement and Concrete Research*	397	Oxygen permeability, rapid chloride penetration, chloride migration, resistivity development, and water sorptivity	[8]
Limestone Calcined Clay Cement and Concrete: A State‐of‐the‐Art Review	Sharma, M.; Bishnoi, S.; Martirena, F.; and Scrivener, K.	2021	*Cement and Concrete Research*	316	Pore structure analysis, chloride ingress resistance, carbonation resistance, alkali–silica reaction (ASR) resistance, sulfate resistance, fire resistance	[19]
Performance of Limestone Calcined Clay Cement (LC3) With Various Kaolinite Contents With Respect to Chloride Transport	Maraghechi, H.; Avet, F.; Wong, H.; Kamyab, H.; and Scrivener, K.	2018	*Materials and Structures*	168	Chloride ingress	[20]
Engineered Cementitious Composites (ECC) With Limestone Calcined Clay Cement (LC3)	Zhang, D.; Jaworska, B.; Zhu, H.; Dahlquist, K.; and Li, V. C.	2020	*Cement and Concrete Research*	164	Pore structure	[21]
High‐Performance Concrete Incorporating Calcined Kaolin Clay and Limestone as Cement Substitute	Du, H. and Pang, S. D.	2020	*Construction and Building Materials*	124	Resistance against moisture and liquid ingress	[22]
Recent progress of utilization of activated kaolinitic clay in cementitious construction materials	Cao, Y. B.; Wang, Y. R.; and Wang, H.	2021	*Composites Part B: Engineering*	107	Sulfate resistance, chloride‐ion penetration	[23]
Experimental Studies on Hydration–Strength–Durability of Limestone–Cement–Calcined Hwangtoh Clay Ternary Composite	Lin, R‐S.; Lee, H‐S.; Han, Y.; Wang, X‐Y.	2021	*Construction and Building Materials*	81	Electrical resistivity, carbonation	[24]
Reinforcement Corrosion in Limestone Flash Calcined Clay Cement‐Based Concrete	Nguyen, Q. D. and Castel, A.	2020	*Cement and Concrete Research*	72	Chloride‐ and carbonation‐induced reinforcing‐bar corrosion	[25]

Sharma et al. [19] presents a state‐of‐the‐art review of LC3 with 316 citations, discussing pore structure analysis, ASR resistance, carbonation resistance, and sulfate resistance. Although the article was recently published; it received a high number of citations. This indicates the high interest if research community in LC3. It was reported from the article that the pH of the pore solution in LC3 has been found to be slightly lower than that of other cements (around 13.8 for OPC and approximately 13.2 for LC3), which suggests that chloride‐induced corrosion is likely to initiate at lower chloride concentrations. This indicates that LC3 may have lower carbonation resistance compared to OPC. However, this issue may be solved by increasing the concrete cover of reinforced elements. Studies by Maraghechi et al. [20] and Zhang et al. [21] focused on chloride ingress and pore structure, respectively, with significant citation counts of 168 and 164, highlighting the importance of transport properties in LC3 research. It was concluded that, when 30% or 50% of clinker was substituted with either calcined clay alone or a blend of calcined clay and limestone, mortar and paste mixtures demonstrated excellent resistance to chloride ion transport, particularly with clays containing around 40% or higher kaolinite content. Additionally, Du and Pang [22] and Cao et al. [23] investigated moisture resistance and sulfate resistance, highlighting the significance of chemical durability concerns. It was noticed that calcined clay‐blended cement has demonstrated excellent durability, including strong resistance to ASRs and sulfate attack, significantly reduced chloride ion penetration, and enhanced protection against aggressive environmental conditions. Research on reinforcement corrosion by Nguyen and Castel [25] and hydration–strength–durability relationships by Lin et al. [24] indicated research interest in long‐term performance and structural integrity, though with comparatively lower citation numbers (72 and 81, respectively). It was found that concrete incorporating flash calcined clay and limestone exhibited performance comparable to conventional Portland cement concrete in long‐term corrosion studies.

Although this article describes publication trends, it is important to critically assess why particular durability aspects dominate the LC3 literature and why others remain understudied. Several interrelated factors likely explain the observed pattern.

First, practical relevance to reinforced concrete durability drives emphasis on chloride ingress and transport. Chloride‐induced reinforcement corrosion is the single most economically significant deterioration mechanism for reinforced concrete in marine and coastal infrastructure; consequently, researchers and funders prioritize studies (e.g., rapid chloride penetration tests, migration tests, and resistivity) that directly address this immediate practical need. Our geographic analysis with high output from countries that face extensive coastal construction and rapid infrastructure growth corroborates this emphasis.

Second, test availability, standardization, and relative speed favor chloride‐related studies. Many chloride transport tests are well standardized or have accepted accelerated protocols that produce publishable results within months, whereas some other durability assessments (e.g., long‐term freeze–thaw with alternate thawing/icing cycles, multiyear combined exposure trials) require long durations, specialized facilities, or complex protocols that are less accessible to many research groups.

Third, geographical and climatic research bias reduces attention to freeze–thaw. A large share of LC3 durability research originates in countries and regions with predominantly warm or temperate climates, where freeze–thaw damage is not a primary concern. As a result, investigators naturally prioritize degradation modes that are locally relevant (chloride, carbonation, sulfate attack), leaving cold‐climate phenomena like freeze–thaw cycles comparatively neglected.

Fourth, perceived material chemistry benefits may discourage some lines of study. Calcined clays consume a portion of available alkalis through pozzolanic reactions, and early experimental evidence suggests LC3 may reduce ASR susceptibility; as a consequence, fewer researchers have prioritized explicit ASR testing relative to other mechanisms. However, this perception risks complacency because ASR outcomes depend strongly on aggregate reactivity, mix design, and local alkali availability factors that vary regionally and require systematic investigation.

Fifth, complexity of combined exposures and extreme conditions makes such studies less common. Realistic combined exposure tests (e.g., simultaneous chloride sulfate or chloride freeze–thaw wetting/drying) are experimentally challenging and resource intensive. They also complicate mechanistic interpretation, which tends to disincentivize early‐stage research teams.

These factors explain why chloride transport and pore‐structure studies lead the LC3 durability literature whereas ASR, freeze–thaw resistance, extreme temperature performance, and combined environmental exposures remain underrepresented.

**Figure 4:**
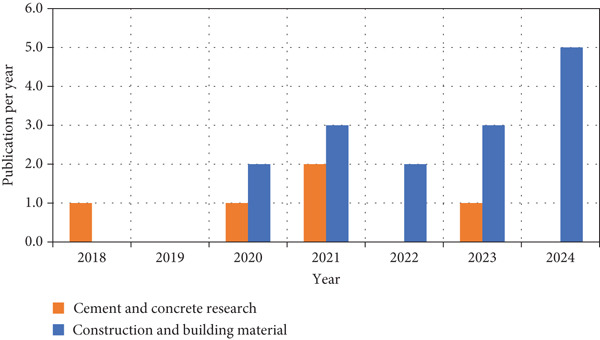
Distribution of publications by journal name per year.

Figure 4 presents the primary sources for publishing research on the durability of LC3. It includes all journals with more than five publications on the topic. The journals were ranked by their performance ratio (PR), which is determined by dividing the total number of citations by the number of publications. Only two journals met this criterion: *Cement and Concrete Research* and *Construction and Building Materials*, with PR values of 193 and 33.4, respectively. The PR results indicate a significant difference between the two journals publishing on durability research on LC3. *Cement and Concrete Research* has a notably high PR of 193, suggesting that its publications on the topic are highly cited, reflecting strong research impact and recognition within the academic community. In contrast, *Construction and Building Materials* has a much lower PR of 33.4, indicating that although it publishes a considerable number of papers, the average citation per paper is significantly lower. This suggests that *Cement and Concrete Research* is a more influential source for high‐impact studies in this field, whereas *Construction and Building Materials* serves as a broader platform, potentially catering to a wider range of research but with lower individual article visibility. The significant PR difference highlights the need for researchers to consider citation impact when selecting journals.

### 3.2. Authors and Countries Evolution

**Figure 5:**
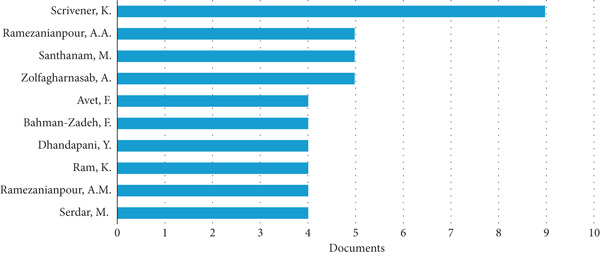
Most productive authors publishing on LC3 durability.

The bar chart in Figure 5 illustrates the most recognized authors in the field of LC3 durability research, measured by the number of documents they have published. The chart lists 10 authors along the vertical axis, with the horizontal axis representing the number of documents, ranging from zero to 10. This visual representation provides a clear comparison of publication output among researchers, highlighting their contributions to the study of LC3 durability.

At the top of the chart, K. Scrivener stands out as the most productive author, with approximately nine documents. This significant output suggests that Scrivener is a leading figure in LC3 durability research, likely contributing foundational studies or reviews that advance the understanding of LC3′s long‐term performance in various environmental conditions. A.M. Ramezanianpour and M. Santhanam have the second‐highest number of publications, each with around six documents.

The next group of authors includes A. Zolfagharnasab, F. Avet, H. Bahman‐Zadeh, Y. Dhandapani, K. Ram, A.M. Ramezanianpour, and M. Serdar, each with three to four documents. This group represents a diverse set of researchers who are actively contributing to the field, though their output is less than that of the top three. For instance, A. Zolfagharnasab and F. Avet, both with around four documents, may be focusing on specific durability aspects like carbonation resistance or the microstructural properties of LC3 that enhance its longevity. Similarly, Y. Dhandapani and K. Ram, each with three documents, might be investigating practical applications or regional variations in LC3 performance, given the global interest in this material. A.M. Ramezanianpour and M. Serdar, also with three documents each, could be exploring complementary areas such as the chemical interactions between LC3 components and aggressive agents like chlorides or sulfates.

**Figure 6:**
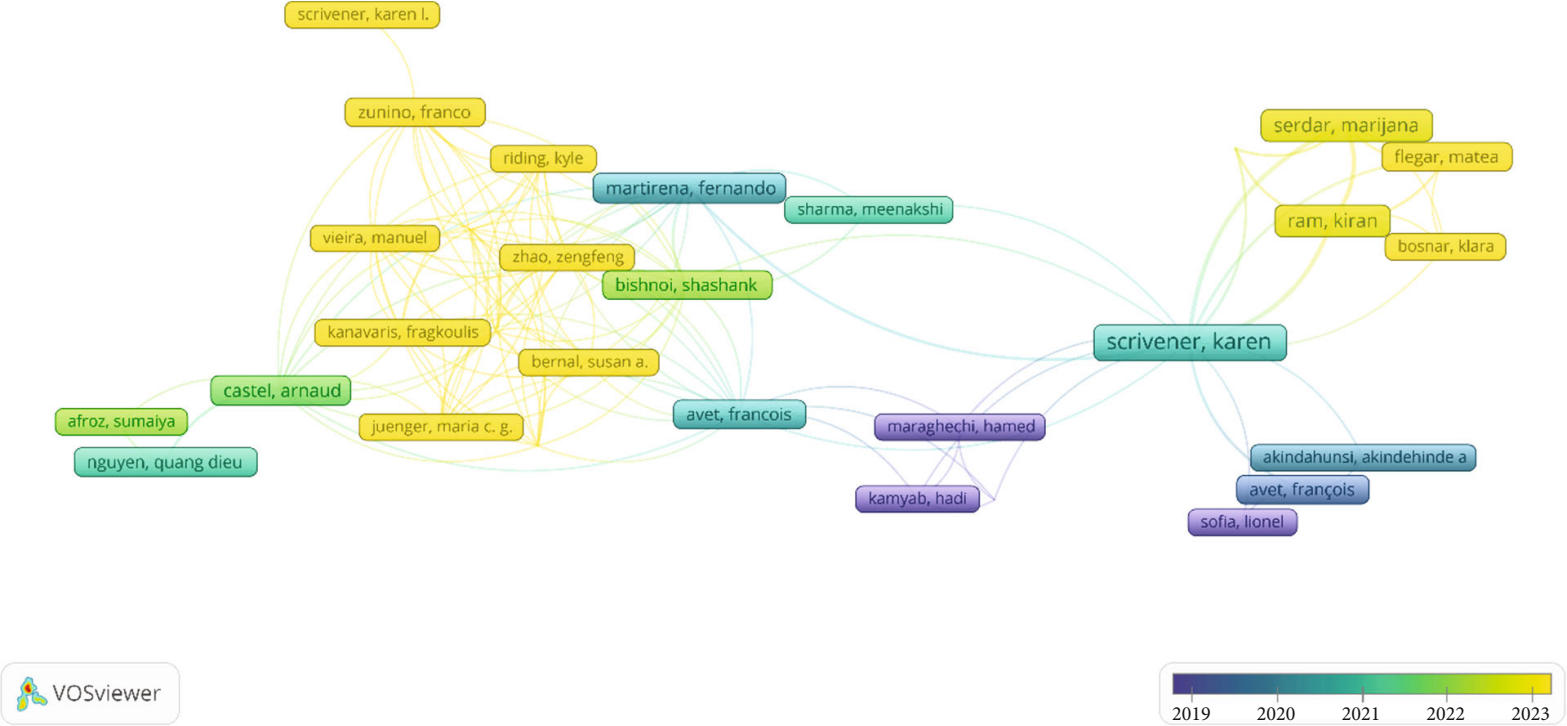
Co‐authorship network visualization for LC3 durability research generated using VOSviewer (version 1.6.20) from Scopus.

The network visualization in Figure 6, generated using VOSviewer, shows the co‐authorship relationships among researchers studying the durability of LC3. Each node represents an author, with the size of the node indicating the number of publications, and the lines (edges) between nodes representing co‐authorship connections. The color gradient, ranging from blue (2019) to yellow (2023), reflects the average publication year of each author′s contributions, providing a temporal perspective on their activity in the field. This network offers insights into the collaborative dynamics, key contributors, and the evolution of research on LC3 durability over time.

At the center of the network, K. Scrivener emerges as a pivotal figure, with the largest node and the most connections, confirming her prominence in LC3 durability research. This aligns with the earlier analysis of Figure 5, where Scrivener was identified as the most recognized author with approximately nine documents. The researcher central position and numerous connections to other researchers, such as F. Martirena, F. Zunino, and F. Avet, suggest that she plays a key role in fostering collaboration, likely through leadership in initiatives like the LC3 Project. The color of the researcher node, leaning toward yellow, indicates that her publications span recent years, with significant activity around 2022–2023, reflecting her ongoing influence in the field.

Several clusters of collaboration are evident in the network. One prominent cluster includes Scrivener, Martirena, Zunino, and Avet, all of whom are closely linked, suggesting a tight‐knit research group. Martirena and Zunino, with nodes colored in shades of green to yellow, have been active between 2020 and 2023, indicating sustained collaboration with Scrivener on recent LC3 durability studies. Avet′s node, slightly smaller but still significant, also shows recent activity around 2022, and his connections extend to other researchers like Sofiane Amziane, reinforcing the collaborative nature of this group. Another cluster includes authors like K. Ram, M. Serdar, and M. Flegar, who are positioned on the right side of the network. Their nodes, colored in yellow, indicate recent publications around 2022–2023, and their connections to Scrivener suggest they may be newer researchers or collaborators contributing to the latest advancements in LC3 durability.

Other notable authors, such as M. Vieira and Q.D. Nguyen, form smaller clusters on the left side of the network. Their nodes, colored in blue and green (around 2019–2020), indicate earlier contributions to LC3 durability research, with fewer connections to the central group. This suggests that their work may have been foundational but less integrated with the more recent collaborative efforts centered around Scrivener. Similarly, authors like S. Bishnoi and H. Maraghechi, positioned near the center but with smaller nodes, show activity around 2020–2021, indicating consistent but less prolific contributions compared to the core group.

The distribution of the network highlights the evolution of LC3 durability research. The presence of blue nodes (2019) on the periphery, transitioning to yellow nodes (2023) closer to the center, suggests that the field has grown and become more collaborative over time, with Scrivener and her network driving much of the recent progress. The density of connections around Scrivener also indicates a highly interconnected research community, which is crucial for advancing a multidisciplinary topic like LC3 durability, where expertise in materials science, chemistry, and engineering must converge.

**Figure 7:**
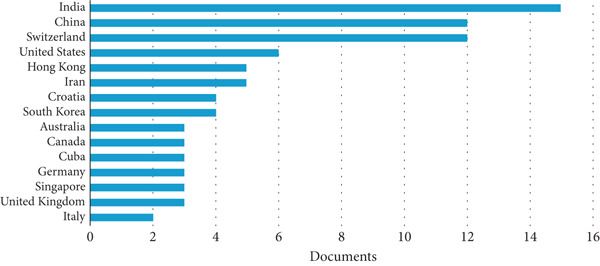
Country‐wise publication output on LC3 durability research (2017–2025) from Scopus.

The bar chart provided in Figure 7 lists the number of documents published on the durability of LC3 by various countries, with a focus on global contributions. The chart includes 14 countries, with the number of documents ranging from two to 15, offering a clear view of research activity in this field.

Starting with the United Kingdom, the chart shows that the country has contributed two documents on LC3 durability. This places the United Kingdom among the lower group of contributors, tied with Germany, Singapore, and Italy, each also having two documents.

Egypt, notably, is not listed among the 14 countries in the chart, which include India, China, Switzerland, the United States, Hong Kong, Iran, Croatia, South Korea, Australia, Cuba, Germany, Singapore, the United Kingdom, and Italy. This absence suggests that Egypt has either not published any documents on LC3 durability or has a publication count below the threshold for inclusion in this chart (likely fewer than two documents). Egypt′s lack of representation is somewhat surprising given its regional context—hot, arid conditions, and proximity to marine environments like the Mediterranean and Red Seas, which could benefit from LC3′s enhanced durability properties, such as resistance to chloride penetration and sulfate attack.

Turning to the “most countries” with the highest publication counts, India leads with approximately 15 documents, followed by China and Switzerland, each with around 10 documents. India′s dominance in LC3 durability research is likely driven by its urgent need for sustainable construction materials, given its rapid urbanization and environmental challenges. The country′s diverse climate, including coastal areas, necessitates robust durability studies, making LC3 a priority. China′s strong showing, with 10 documents, reflects its massive cement industry responsible for over half of the global production and its push toward sustainability. Chinese researchers, potentially including those like Yu Zhang, are likely exploring LC3′s durability in industrial and infrastructure applications, addressing challenges like carbonation and freeze–thaw cycles in varied climates. Switzerland, also with 10 documents, owes its prominence to the leadership of K. Scrivener at EPFL, a hub for LC3 research. As seen in the co‐authorship network, Scrivener′s extensive collaborations amplify Switzerland′s output, focusing on fundamental durability aspects like pore structure and chemical resistance, which are critical for LC3′s global adoption.

The next group of high‐output countries includes the United States, Hong Kong, and Iran, each with five to six documents. The United States contribution likely stems from academic and industry efforts to explore sustainable cements, though its focus may be broader than LC3 alone. Hong Kong′s research might address durability in high‐density urban settings, whereas Iran′s involvement could be driven by the need for durable materials in its harsh environmental conditions, such as high salinity in the Persian Gulf region. These countries, although not at the top, still play a significant role in the global research landscape for LC3 durability.

### 3.3. Keywords

**Figure 8:**
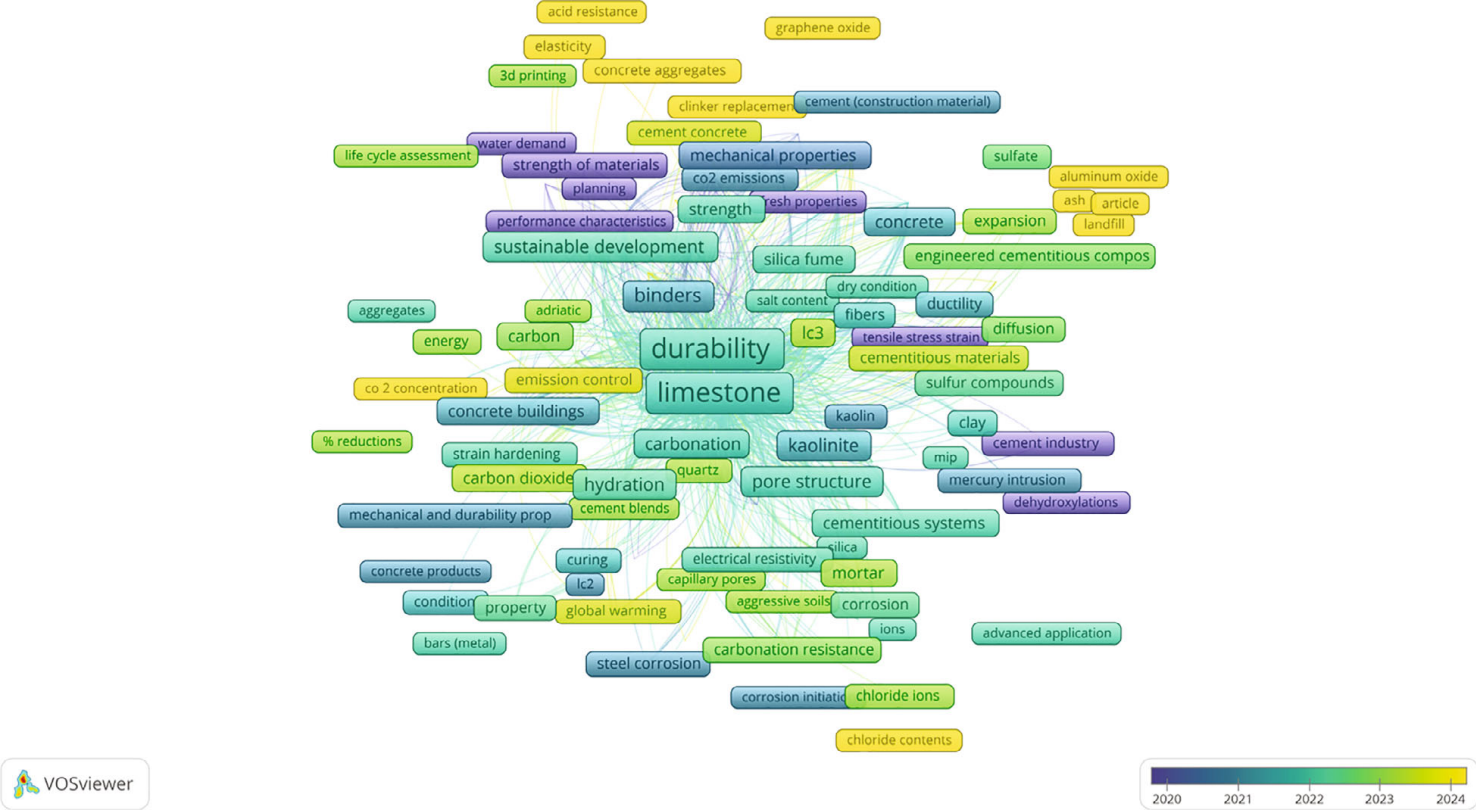
Keyword co‐occurrence network for LC3 durability research visualized using VOSviewer (version 1.6.20) from Scopus.

The VOSviewer network visualization in Figure 8 shows the co‐occurrence of keywords in research on the durability of LC3. Each node represents a keyword, with its size indicating the frequency of occurrence, and the lines between nodes showing how often keywords appear together in publications. The color gradient, ranging from blue (2020) to yellow (2024), reflects the average publication year of the keywords, offering a temporal perspective on research trends. The central keyword, “durability limestone calcined clay cement,” connects to various clusters, revealing the main themes and focus areas in LC3 durability studies. By analyzing this network, we can identify key research areas and potential gaps in durability testing, particularly in areas that are underrepresented or missing.

The network reveals several prominent clusters of keywords around the central theme of LC3 durability. One major cluster, primarily in green and yellow (2022–2024), includes terms like “durability,” “LC3,” “compressive strength,” “microstructure,” “chloride ions,” “carbonation,” and “pore structure.” These keywords indicate a strong focus on fundamental durability aspects, such as resistance to chloride penetration (important for marine environments) and carbonation (which affects long‐term performance in CO_2_‐rich environments). The emphasis on “microstructure” and “pore structure” suggests that researchers are investigating how LC3′s internal composition particularly the refined pore network due to calcined clay enhances its durability. The recent publication dates (yellow nodes) show that these topics remain a priority, reflecting ongoing efforts to understand LC3′s performance under aggressive conditions.

Another cluster, with nodes in blue and green (2020–2022), includes keywords like “binders,” “hydration,” “mechanical properties,” “sustainability,” and “cement.” This cluster highlights the early focus on LC3′s material properties, such as how its hydration process (involving clinker, calcined clay, and limestone) contributes to mechanical strength and sustainability. Terms like “sustainability” and “global warming” indicate that LC3′s environmental benefits, such as reduced CO_2_ emissions, are often studied alongside durability, positioning it as a viable alternative to traditional Portland cement. However, this cluster is less directly tied to specific durability tests, focusing more on the material′s overall performance and environmental impact.

A smaller cluster on the right, with keywords like “permeability,” “diffusion,” “mercury intrusion,” and “cement industry,” suggests research into LC3′s transport properties, which are critical for durability. Permeability and diffusion directly influence how aggressive agents like water, chlorides, or sulfates penetrate the material, affecting its long‐term performance. The presence of “mercury intrusion” (a method to measure pore size distribution) aligns with the focus on pore structure in the main cluster, indicating that researchers are using advanced techniques to study LC3′s durability at a microstructural level. These keywords, mostly in green (2021–2022), show that this area has received attention but may not be as dominant in more recent studies (fewer yellow nodes).

Despite the comprehensive coverage of many durability aspects, several gaps in durability testing can be inferred from the network. First, there is a noticeable lack of keywords related to freeze–thaw resistance, a critical durability factor in cold climates. LC3′s performance in regions with repeated freezing and thawing cycles, which can cause cracking and degradation due to water expansion, appears underexplored. This gap is significant because LC3 is being promoted as a global solution, yet its durability in colder climates, such as those in parts of the United States, Canada, or Northern Europe, is not well‐represented in the keyword network. Testing LC3 under freeze–thaw conditions, especially with deicing salts, could be a crucial area for future research to ensure its applicability in diverse environments.

Second, the network lacks keywords specifically addressing ASR, a durability concern in concrete where reactive aggregates react with alkalis in the cement, leading to expansion and cracking. Although “aggregates” appear as a keyword, it is not strongly linked to ASR, suggesting that this aspect of LC3 durability has not been a primary focus. Given that LC3 contains calcined clay, which can reduce ASR by consuming alkalis during its pozzolanic reaction, this gap is surprising. Research into LC3′s ability to mitigate ASR, especially in regions with reactive aggregates (e.g., parts of Australia or the Middle East), could further validate its durability and expand its practical applications.

Third, there is a limited focus on long‐term durability under combined environmental exposures, such as simultaneous chloride and sulfate attack, which is common in coastal or industrial settings. Although “chloride ions” and “sulfate” appear as keywords, they are not strongly interconnected, and there is no mention of combined exposure tests. In real‐world conditions, concrete often faces multiple aggressive agents at once, and understanding how LC3 performs under such synergistic effects is critical for its adoption in harsh environments, such as marine structures in sulfate‐rich waters (e.g., the Persian Gulf). This gap suggests a need for more comprehensive durability tests that simulate real‐world conditions more closely.

Additionally, the network shows a lack of keywords related to durability in extreme temperatures, such as high‐temperature exposure (e.g., fire resistance) or very low temperatures beyond freeze–thaw cycles. LC3′s behavior under thermal stress, which can affect its phase composition and mechanical properties, is not well represented. For instance, high temperatures can dehydrate cement phases, whereas calcined clay′s thermal stability might offer advantages that are currently underexplored.

## 4. Proposed Methods to Address Research Gaps

### 4.1. Freeze–Thaw Resistance

To evaluate LC3′s performance in cold climates, researchers should apply standard cyclic freezing and thawing tests, such as ASTM C666 (Procedure A or B) [26], with continuous monitoring of mass loss, dynamic modulus, and surface scaling. Microstructural evaluation using and scanning electron microscopy (SEM) before and after cycling can help correlate pore structure refinement with freeze–thaw durability.

### 4.2. ASR

LC3′s pozzolanic nature and lower pore‐solution alkalinity suggest potential ASR mitigation, but this requires verification through ASTM C1260 (accelerated mortar bar) [27] and ASTM C1293 (concrete prism) [28] tests using different reactive aggregates. Furthermore, microstructural analysis via SEM can identify ASR gel formation and assess crack propagation at the aggregate–paste interface.

### 4.3. Combined Environmental Actions

To simulate real‐world exposure, future work should integrate multiple deterioration mechanisms. For example, chloride ingress under cyclic wetting–drying conditions can be coupled with sulfate exposure to replicate marine environments.

### 4.4. Elevated Temperature Performance

Limited studies exist on LC3 at high temperatures. Tests following ASTM E119 (fire resistance) [29] can be used to assess mechanical and microstructural degradation up to 800°C. Thermogravimetric analysis (TGA) and differential scanning calorimetry (DSC) should be applied to evaluate dehydration and phase transformations.

### 4.5. Long‐Term Field Validation

Finally, controlled outdoor exposure sites in different climatic regions (e.g., tropical, marine, and cold continental) should be established to validate laboratory findings and develop empirical models linking exposure severity to degradation rate.

## 5. Summary and Conclusions

This bibliometric study on the durability of LC3 adopts the methodology of Mañosa et al. [10] and analyzes 21 publications retrieved from Scopus and WoS databases covering the period 2017–2025. Using MS Excel and VOSviewer, the study evaluated publication trends, author productivity, journal performance, and country contributions. The results show a consistent rise in research output, from two papers per year in 2017–2019 to a peak of 14 publications in 2023–2024, reflecting the growing interest in LC3 durability. India leads with 15 documents, followed by China and Switzerland (10 each), whereas the United Kingdom contributed two and Egypt was not represented. The most influential journals are *Cement and Concrete Research* (PR = 193) and *Construction and Building Materials* (PR = 33.4). The most cited study, Dhandapani et al. [8], has 397 citations, confirming LC3′s superior resistance to chloride ingress and permeability. Another key contribution, Sharma et al. [19], with 316 citations, provides an in‐depth review of LC3′s microstructural and chemical durability mechanisms. The keyword analysis revealed that current research predominantly targets chloride transport, carbonation, and microstructural development, whereas other durability aspects such as freeze–thaw resistance, ASR, combined environmental exposures, and high‐temperature performance remain underexplored.

Future research should focus on the following:

• Evaluating freeze–thaw resistance and ASR under varied environmental conditions.
• Investigating combined degradation mechanisms, such as chloride–sulfate or chloride–freeze–thaw interactions.
• Assessing LC3 performance under elevated temperatures (e.g., fire resistance, thermal decomposition).
• Conducting long‐term field exposure tests in diverse climates to validate laboratory results.
• Promoting international collaborations and regional studies (especially in Africa and the Middle East) to enhance the global adoption of LC3 in sustainable construction.

## Conflicts of Interest

The authors declare no conflicts of interest.

## Author Contributions

Xiangming Zhou participated in the conceptualization and review of the article, Rabee Shamass participated in the conceptualization and review of the article, and Ayman Shamseldein participated in writing the first draft and investigation of the article.

## Funding

This research is part of the ISPF Early Career Fellowship Scheme–Egypt, sponsored by the British Council through the International Science Partnerships Fund. It falls under the subproject titled “Nurturing Early Career Fellows in Climate Resilient and Sustainable Built Environment Research Agenda,” with project code 13003100.

## Data Availability

The datasets used and/or analyzed during the current study are available from the corresponding author on reasonable request.
